# Correlation between patient age at total hip replacement surgery and lifeexpectancy

**DOI:** 10.1590/1413-785220152306148609

**Published:** 2015

**Authors:** Carlos Roberto Schwartsmann1, Leandro de Freitas Spinelli, Leonardo Carbonera Boschin, Anthony Kerbes Yépez, Marcus Vinicius Crestani, Marcelo Faria Silva

**Affiliations:** 1 Universidade Federal de Ciências da Saúde de Porto Alegre e Santa Casa de Misericórdia de Porto Alegre, Ortopedia e Traumatologia, Porto Alegre, RS, Brasil; 2 Santa Casa de Misericórdia de Porto Alegre, Ortopedia e Traumatologia, Cirurgia do Quadril, Porto Alegre, RS, Brasil; 3 Universidade Federal de Ciências da Saúde de Porto Alegre, Fisioterapia, Porto Alegre, RS, Brasil

**Keywords:** Arthroplasty, replacement, hip. Follow-up studies. Survival

## Abstract

Total hip arthroplasty (THA) is one of the most cost-effective hip surgeries among orthopedic procedures. We conducted an extensive literature review with 5,394 papers regarding survival rates after THA. We searched PubMed, Embase and the Cochrane library from January 1^st^, 1970 to July 31^th^, 2014 looking for all citations about total hip arthroplasty with a long term follow-up (longer than 10 years). The criteria were rigorous: no loss of follow-up, and follow-up more than 10 years. The authors should have known the complete history of patients (whether the patient is alive or dead). Considering the criteria, we found only 15 papers. To evaluate the relationship between follow-up and survival, a linear regression analysis was applied. Considering the papers analysed, and applying the search criteria, we obtained a mean age for the patients above 57.5 years. The chance of survival at 15 years was 57.6%, at 20 years it was 34.6% and at 25 years it is only 11.6%. The relationship between follow-up and survival was significantly linear (p <0.001). Only 11.6% of patients undergoing THA will be still alive 25 years after the surgical procedure. **Level of Evidence I, Prognostic Study.**

## INTRODUCTION

Total hip arthroplasty (THA) is one of the most cost-effective surgeries among orthopaedic procedures. Several authors have suggested that patients undergoing this procedure have a higher survival rate than the general population.^1^ ^7^ However, there are still no publications presenting a clear and unequi-vocal form of the survival of patients after the performance of total hip arthroplasty.

In order to find the answer for the survival of patients after the performance of total hip arthroplasty, we conducted an ex-tensive literature review. After careful review, we selected only papers including a long follow-up (mean follow-up of more than 10 years), known mortality, and in which there was no loss of follow-up regarding the patients.

## PATIENTS AND METHODS

A review of the literature was conducted. We searched the computerised databases PubMed, Embase and the Cochrane library from January 1^st^, 1970 to July 31^th^, 2014 looking for all citations about total hip arthroplasty or hip replacement, and long follow-up (longer than 10 years). All papers with a mean follow-up smaller than 10 years, and papers presenting lost of follow-up were excluded from the study.

Most of the initially found papers present significant lack of im-portant information, which compromises the statistical analysis. It was fundamental that the authors know how many patients had died or continued to live at the end of the follow-up. The searching words were: hip arthroplasty or/and hip replacement and follow-up. The search is very sensitive. We found 27,694 papers on the initial search. After a preliminary analysis, 5394 papers were selected. We excluded biomechanical studies, reviews of any kind, surveys, laboratory or radiologic, and ex-perimental animal studies. Then the eligible publications that met the inclusion criteria were evaluated.

Initially, we included only papers with minimum follow-up of 10 years, in which the authors cited the number of deaths, and in which there was no lost of follow-up higher than 10%. In the first stage, the authors reviewed all abstracts of articles on THA with a follow-up over 10 years. At this stage, a total of 124 out of 5394 papers were selected 

 In the second stage, these 124 articles were read in full and their references were reviewed to identify other papers that might have been lost in the first selection. At the end of the second phase, we found only 44 articles with less than 10% follow-up loss, totalling 13,357 patients. Of these 44 papers, only 15 presented no lost of follow-up, and the authors therefore knew how many patients had died with a minimum follow-up of 10 years. The phrase "no loss of follow-up" should be present somewhere in the text.

After this selection, the mean follow-up, patient survival and projections for 15, 20 and 25 years were calculated. Based on this information, an equation that best describes the relationship between these factors was obtained. In performing this calcu-lation, we consider that the boards of living populations of the analysed papers were the same, or very similar, as was the distribution of deaths during follow-up. Thus, it was possible to use the average as a representation of global behaviour. To evaluate the relationship between follow-up and survival, a linear regression analysis was applied, considering the mean age, time and mortality.

## RESULTS

**Figure 1:**
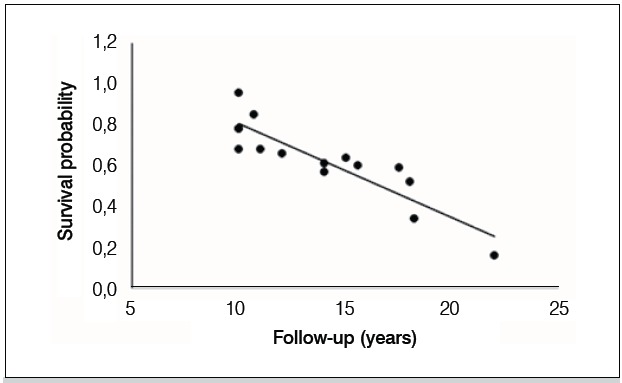
Survival probability versus follow-up of the patients curve.

Table 1 presents the life expectancy of patients in the 15 se-lected papers^8-22^ in which the authors have not lost any case to follow-up and knew exactly how many had died during the period. Figure 1 shows the studies through a curve of survival probability versus follow-up of the patients. The chance of patient survival at 15 years is 57.6%, at 20 years is 34.6% and at 25 years is only 11.6%. The relationship between follow-up and survival was significantly linear ( *p <0.001* ); with each following year, the probability of survival decreased by 4.6%. When ad-justed for age, the association remained significant ( *p <0.001* ).

## DISCUSSION

Although there are numerous papers that have reported survival of THA, few include longer follow-up, without any loss of pa-tients. The total hip arthroplasty decisively modifies the quality of life of patients. However, it is not a goal in the surgical indication to increase the survival of patients. Numerous authors, analy-sing statistics with long-lasting follow-up show that the survival curves of patients with THA are better than the corresponding analysed populations.^1^ ^7^

Lie et al.^1^ observed that after a mean follow-up of 8 years, the mortality of patients with THA was 25% versus 30% in the cor-responding Norwegian population. The authors propose several hypotheses to explain this fact and motivate a discussion. Visuri et al.^6^ tried to explain this event by the selective process that these patients undergo after the indication for elective surgery. Barrett et al.^4^ believe that patients return to having comple-te mastery over themselves and this determines a favourable change of behaviour. However, the calculation of survival in patients with THA is affected by a very large number of variants and is difficult to assess reliably.

However, this expectation of survival can be considered a very important and almost unquestionable factor in planning the surgery. If the patient is aged 90 years or over, has rheumatoid arthritis, pulmonary metastatic cancer, Paget, osteoporosis, etc., that will influence the surgeon about the type of prosthesis, type of fixation, femoral head size, etc.

The reliability of the calculation of survival of patients with THA may be questioned if we do not have follow-up of all patients undergoing surgery. This would be essential, but in practice the literature presents losses of 5-52%, depending on the years in which the patients are followed.^23^ ^26^

Toni et al.^26^ reported that the loss in his series reached 51.5% af-ter 11 years of follow-up. For this reason, large loss of follow-up can significantly compromise the survival curves of arthroplasty patients, the reviews and own results, as they become virtually unreliable. This does not occur in smaller segments because we have most of the events with patients environmentally controlled. Berstock et al.^27^, in a systematic review of 30-days and 90-days mortality after total hip arthroplasty, concluded that the most commonly identified risk factors for early mortality are increasing age, male gender, and co-morbidity conditions, particularly cardiovascular disease.

**Table 1:**
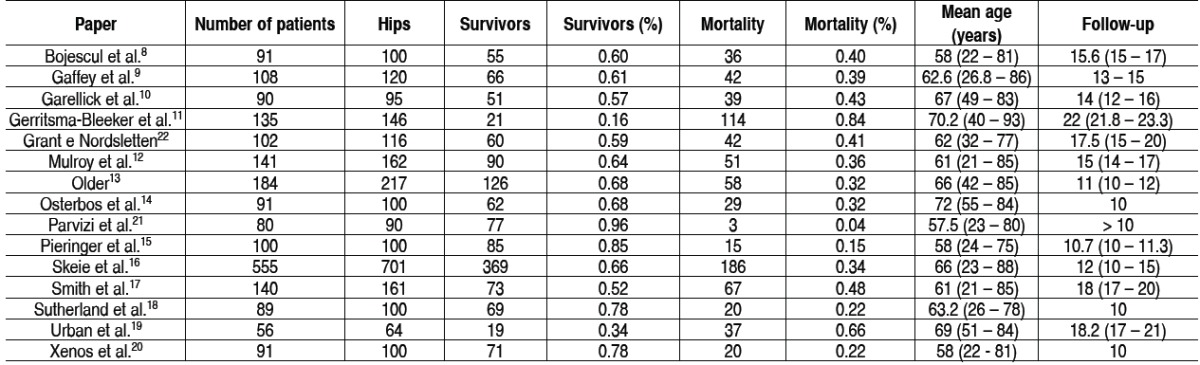
Survival expectancy of the selected papers.

However, in the literature, most analyses of the results com-monly assume that patients with subsequent losses have the same evolution as those who are regularly reviewed.^28^ ^30^ Few admit that those cases in which the segments have been lost are inferior to those that were regularly analysed.^23^ ^25^

In this study, the first selection criterion considered how long patients should be followed in order to offer a reliable statistical curve. Of course the answer would be the longest possible follow-up. However, there are very few studies with 20, 25 or 30 years of follow-up. We have established that all studies with less than 10 years follow-up should not be included in this review. The second criterion evaluated was the loss to follow-up. First, we analysed papers with loss to follow-up of less than 10%. After reviewing 5394 articles on THA, we found only 44 articles with over 10 years of follow-up and less than 10% loss. Howe-ver, if the following criteria demand that the authors say that there was no loss of follow-up, we found only 15 articles. After this selection, the index of mortality and survival rates of each paper were calculated according to the average follow-up time, and it was observed that the survival of the population after 25 years was 11.6%.

Another interesting topic for discussion is the age of the pa-tients. Nine from 15 of the assessed articles include patients operated at the age of less than 30 years, and this is a bias. The research used the mean age of the selected patients. In four papers, the mean age was about 58 years.^8^ ^15^ ^20^ ^21^ Guerritsma--Bleeker et al.^11^ with 22 years of follow-up, and a mean age of 70.2 years found a mortality of 84%. Bojescul et al.^8^ with 15.6 years of follow-up, and a mean age of 58 years, found a morta-lity of only 40%. Since, as the mean age of the group becomes higher, as higher we found the mortality ratio.

## FINAL REMARKS

Considering the follow-up of more than 10 years, and the no lost of follow-up criteria, and the mean age of patients of more than 57.5 years, only 11.6% of patients undergoing THA will remain alive 25 years after the surgical procedure. 

## References

[B1] Lie SA, Engesaeter LB, Havelin LI, Gjessing HK, Vollset SE (2000). Mortality after total hip replacement: 0-10-year follow-up of 39,543 patients in the Norwegian Arthroplasty Register. Acta Orthop Scand.

[B2] Holmberg S (1992). Life expectancy after total hip arthroplasty. J Arthroplasty.

[B3] Ritter MA, Albohm MJ, Keating EM, Faris PM, Meding JB (1998). Life expectancy after total hip arthroplasty. J Arthroplasty.

[B4] Barrett J, Losina E, Baron JA, Mahomed NN, Wright J, Katz JN (2005). Survival following total hip replacement. J Bone Joint Surg Am.

[B5] Lindberg H, Carlsson AS, Lanke J, Horstmann V (1984). The overall mortality rate in patients with total hip arthroplasty, with special reference to coxarthrosis. Clin Orthop Relat Res.

[B6] Visuri T, Pulkkinen P, Turula KB, Paavolainen P, Koskenvuo M (1994). Life expectancy after hip arthroplasty. Case-control study of 1018 cases of primary arthrosis. Acta Orthop Scand.

[B7] Whittle J, Steinberg EP, Anderson GF, Herbert R, Hochberg MC (1993). Mortality after elective total hip arthroplasty in elderly Americans. Age, gender, and indication for surgery predict survival. Clin Orthop Relat Res.

[B8] Bojescul JA, Xenos JS, Callaghan JJ, Savory CG (2003). Results of porous-coated anatomic total hip arthroplasty without cement at fifteen years: a concise follow--up of a previous report. J Bone Joint Surg Am.

[B9] Gaffey JL, Callaghan JJ, Pedersen DR, Goetz DD, Sullivan PM, Johnston RC (2004). Cemen-tless acetabular fixation at fifteen years. A comparison with the same surgeon's results following acetabular fixation with cement. J Bone Joint Surg Am.

[B10] Garellick G, Herberts P, Strömberg C, Malchau H (1994). Long-term results of Charn-ley arthroplasty. A 12-16-year follow-up study. J Arthroplasty.

[B11] Gerritsma-Bleeker CL, Deutman R, Mulder TJ, Steinberg JD (2000). The Stanmore total hip replacement. A 22-year follow-up. J Bone Joint Surg Br.

[B12] Mulroy WF, Estok DM, Harris WH (1995). Total hip arthroplasty with use of so-called second-generation cementing techniques. A fifteen-year-average follow-up study. J Bone Joint Surg Am.

[B13] Older J (2002). Charnley low-friction arthroplasty: a worldwide retrospective review at 15 to 20 years. J Arthroplasty.

[B14] Oosterbos CJ, Rahmy AI, Tonino AJ, Witpeerd W (2004). High survival rate of hydro-xyapatite-coated hip prostheses: 100 consecutive hips followed for 10 years. Acta Orthop Scand.

[B15] Pieringer H, Auersperg V, Griessler W, Böhler N (2003). Long-term results with the cemen-tless Alloclassic brand hip arthroplasty system. J Arthroplasty.

[B16] Skeie S, Lende S, Sjøberg EJ, Vollset SE (1991). Survival of the Charnley hip in coxarthrosis. A 10-15-year follow-up of 629 cases. Acta Orthop Scand.

[B17] Smith SE, Estok DM 2nd, Harris WH (2000). 20-year experience with cemented pri-mary and conversion total hip arthroplasty using so-called second-generation cementing techniques in patients aged 50 years or younger. J Arthroplasty.

[B18] Sutherland CJ, Wilde AH, Borden LS, Marks KE (1982). A ten-year follow-up of one hundred consecutive Müller curved-stem total hip-replacement arthroplasties. J Bone Joint Surg Am.

[B19] Urban JA, Garvin KL, Boese CK, Bryson L, Pedersen DR, Callaghan JJ, (2001). Ceramic-on-polyethylene bearing surfaces in total hip arthroplasty. Seventeen to twenty-one-year results. J Bone Joint Surg Am.

[B20] Xenos JS, Callaghan JJ, Heekin RD, Hopkinson WJ, Savory CG, Moore MS (1999). The porous-coated anatomic total hip prosthesis, inserted without cement. A prospective study with a minimum of ten years of follow-up. J Bone Joint Surg Am.

[B21] Parvizi J, Sullivan T, Duffy G, Cabanela ME (2004). Fifteen-year clinical survivorship of Harris-Galante total hip arthroplasty. J Arthroplasty.

[B22] Grant P, Nordsletten L (2004). Total hip arthroplasty with the Lord prosthesis. A long--term follow-up study. J Bone Joint Surg Am.

[B23] Murray DW, Britton AR, Bulstrode CJ (1997). Loss to follow-up matters. J Bone Joint Surg Br.

[B24] Fender D, Harper WM, Gregg PJ (2000). The Trent regional arthroplasty study. Expe-riences with a hip register. J Bone Joint Surg Br.

[B25] Wildner M (1995). Lost to follow-up. J Bone Joint Surg Br.

[B26] Toni A, Stea S, Bordini B, Traina F (2002). Lost to follow-up in a hip prosthesis register. Experience of R.I.P.O. Acta Orthop Scand Suppl.

[B27] Berstock JR, Beswick AD, Lenguerrand E, Whitehouse MR, Blom AW (2014). Morta-lity after total hip replacement surgery: A systematic review. Bone Joint Res.

[B28] Dorey F, Amstutz HC (1989). The validity of survivorship analysis in total joint arthro-plasty. J Bone Joint Surg Am.

[B29] Herberts P, Malchau H (2000). Long-term registration has improved the quality of hip replacement: a review of the Swedish THR Register comparing 160,000 cases. Acta Orthop Scand.

[B30] Lucht U (2000). The Danish Hip Arthroplasty Register. Acta Orthop Scand.

